# Exotic dried fruits caused *Salmonella* Agbeni outbreak with severe clinical presentation, Norway, December 2018 to March 2019

**DOI:** 10.2807/1560-7917.ES.2021.26.14.2000221

**Published:** 2021-04-08

**Authors:** Tone Bjordal Johansen, Lin T Brandal, Emily MacDonald, Umaer Naseer, Pawel Stefanoff, Margrethe Hovda Røed, Turid M Berglund, Gro S Johannessen, Bjarne Bergsjø, Line Vold, Heidi Lange

**Affiliations:** 1 Norwegian Institute of Public Health, Oslo, Norway; 2 European Public Health Microbiology Training Programme (EUPHEM), European Centre for Disease Prevention and Control (ECDC), Stockholm, Sweden; 3 The Norwegian Food Safety Authority, Oslo, Norway; 4 Norwegian Veterinary Institute, Oslo, Norway

**Keywords:** *Salmonella* Agbeni, outbreak, dried mixed fruit, whole genome sequencing, ready-to-eat products

## Abstract

We describe an outbreak of *Salmonella* Agbeni sequence type (ST)2009 infections in Norway. Between 31 December 2018 and 16 March 2019, 56 cases (33 female and 23 male; median age: 50 years, range: 2–91) were reported, of which 21 were hospitalised. Cases were defined as people living in Norway, with laboratory-confirmed infection with *S*. Agbeni ST2009 and cluster type (CT)2489, reported between 31 December 2018 and 30 March 2019. We conducted a case–control study, with three controls per case (matched by age, sex and municipality), using the Norwegian National Registry. Cases were more likely to have consumed a commercial mix of dried exotic fruits than controls (cases = 8, controls = 31; odds ratio: 50; 95% confidence interval: 3–2,437). The outbreak strain was confirmed by whole genome sequencing (WGS) and was isolated from the fruit mix consumed by cases, resulting in withdrawal from the market on 6 March 2019.The fruit mix consisted of fruits from different countries and continents. It was packed in Italy and distributed to several European countries, including Norway. However, no other countries reported cases. This outbreak highlights that dried fruits could represent a risk in terms of food-borne infections, which is of particular concern in ready-to-eat products.

## Background

Non-typhoid salmonellosis is a gastrointestinal infection characterised by diarrhoea, nausea and occasionally vomiting and fever. In 2017, 20 confirmed salmonellosis cases per 100,000 population were reported in the European Union (EU), making it the second most commonly reported food-borne infection [1]. In Norway, it is mandatory to report all cases of salmonellosis to the Norwegian Surveillance System for Communicable Diseases (MSIS), and the medical microbiology laboratories submit *Salmonella* isolates to the National Reference Laboratory for Enteropathogenic Bacteria (NRL) at the Norwegian Institute of Public Health (NIPH) for confirmation and molecular epidemiological surveillance by whole genome sequencing (WGS). The incidence rate was 18 per 100,000 inhabitants in 2018 [2].

The majority of cases are travel-related, as Norway has few known domestic reservoirs [3]. The dominating serotypes detected are *Salmonella* Typhimurium and *Salmonella* Enteritidis. Outbreaks involving different serovars of *Salmonella* are observed irregularly in Norway, with four national outbreaks reported in 2018 [2-4].

## Outbreak detection

On Tuesday 12 February 2019, the NRL identified a cluster of four *S.* Agbeni isolates, identical by WGS. Previously, this rare serotype of *Salmonella* had only been reported from a few sporadic cases in Norway and from a few outbreaks in the United States (US) and Canada [5-7]. The cases resided in different municipalities in Norway. The following week, three more cases were detected. The initial interviews indicated a dried fruit mix product as the possible source of the outbreak. The NIPH initiated an outbreak investigation in collaboration with the Norwegian Food Safety Authority (NFSA) and the Norwegian Veterinary Institute (NVI) to identify the source of the outbreak and implement control measures.

This article describes the outbreak investigation and public health measures, and the finding that consumption of a ready-to-eat snack product of dried exotic fruits caused the outbreak of *S.* Agbeni in Norway.

## Methods

### Case definition

We defined a case as a person living in Norway with laboratory-confirmed infection with *S*. Agbeni, sequence type (ST)2009 and cluster type (CT)2489, reported between 31 December 2018 and 30 March 2019.

### Epidemiological investigations

The local food safety authorities interviewed the initial cases using a standardised 19-page *Salmonella*-specific trawling questionnaire, in order to identify the probable source of infection. The questionnaire included questions regarding symptoms, date of onset, travel history and consumption of a range of foods during the week before onset of symptoms. We used Excel 2016 to describe the cases and summarise the results of the interviews. Missing information from trawling interviews was omitted from the analysis.

### Case–control study

On 6 March, we initiated a case–control (CC) study to confirm the preliminary hypothesis on the likely source of the outbreak and explore the possible role of other exposures and confounding factors. For the case–control study, we used the outbreak case definition. Cases already interviewed with the trawling questionnaire were excluded. Three controls per case were randomly selected from the Norwegian National Registry, individually matched to the cases by age, sex and municipality. We interviewed cases and controls by phone regarding consumption of products identified through the trawling questionnaires. Consumption of dried fruits, nuts, chicken and different spices was recorded during the week preceding the onset of symptoms in cases or the week preceding the interview in controls. Exclusion criteria for controls were travel abroad or diarrhoea (three or more loose stools per day for at least 1 day) in the last 4 weeks before the interview.

We calculated odd ratios (ORs) with 95% confidence intervals (CIs) for common exposures in StataSE/15.0 by univariable unmatched analysis. For all questions about food consumption, the cases and controls could reply ‘yes’, ‘probably yes’, ‘probably no’, ‘no’ and ‘unsure’, regarding whether or not they consumed each food item. For the analysis, ‘yes’ and ‘probably yes’ were coded as yes and ‘probably no’ and ‘no’ were coded as no. ‘Unsure’ was reported as missing.

### Additional investigations

Following trawling and case–control interviews, an electronic questionnaire with photographs of dried fruit and nut products with packaging labels, including the suspected fruit mix (see Supplementary Figure S1), were sent to cases. Cases that were identified late in the investigation and therefore not included in the interviews described previously were asked to complete a shorter version of this electronic questionnaire. They were also briefly interviewed by phone and asked for clinical symptoms, date of disease onset and travel history. In addition, we collected grocery store receipts from cases based on information from the interviews.

### Microbiological investigations

#### Human cases

During the outbreak period, rapid serotyping of isolates was conducted using standard methods to identify the serovar *S*. Agbeni [8]. WGS was performed on all *Salmonella* isolates received at the NRL to determine the ST [9] and CT based on core-genome multilocus sequence typing using the EnteroBase scheme v2 [10] run in SeqSphere + , version 5.1.0 (Ridom GmbH, Münster, Germany). Allele calling procedure with a minimum accepted BLAST identity of 80%, no BLASTp search, frame-shift detection turned on and independent SeqSphere ^+^  allele numbering nomenclature was applied. The allelic profiles of the isolates were visualised as a minimum spanning tree using the parameter ‘pairwise ignoring missing values’ [11]. A CT was defined as a single-linkage threshold of ≤ 7 alleles. The sequences were submitted to the European Nt Archive under the access numbers ERR3173399, ERR4143502, ERR4143569 and ERR4145222.

#### Food samples

The NFSA collected food samples from nine cases that had leftovers of suspected products (dried fruit mix, nuts and spices) or directly from their local retailer. Among the collected samples, three samples of dried fruit mix collected from cases in different parts of Norway were found to originate from the same batch. Microbiological analysis was performed at the NVI according to standard methodology for food analysis [12,13]. Following preliminary results from these tests, additional samples from the Norwegian fruit and vegetable importer and distributor were analysed in a private laboratory, and isolates were sent for confirmation to the NRL for non-human *Salmonella* at NVI. All isolates confirmed as *S*. Agbeni were sent to the NRL to be compared with the outbreak strain by WGS.

### Trace-back investigations

Based on interviews and microbiological investigations, the product was traced back to the distributor. The NFSA contacted the Norwegian distributor on 6 March 2019, to conduct a trace-back investigation of the food source. The suspected retail company was located in a European country, and an alert notification was sent through the European Commission Food and Feed Safety Alerts (RASFF 2019.0905) on 7 March, to notify the authorities about the suspected product and to request more information.

### Ethical statement

Ethical approval was not required, as outbreak investigations are covered under national legislation. Cases were asked for consent to participate at the start of the interviews.

## Results

### Epidemiological investigations

#### Descriptive epidemiology

**Figure 1:**
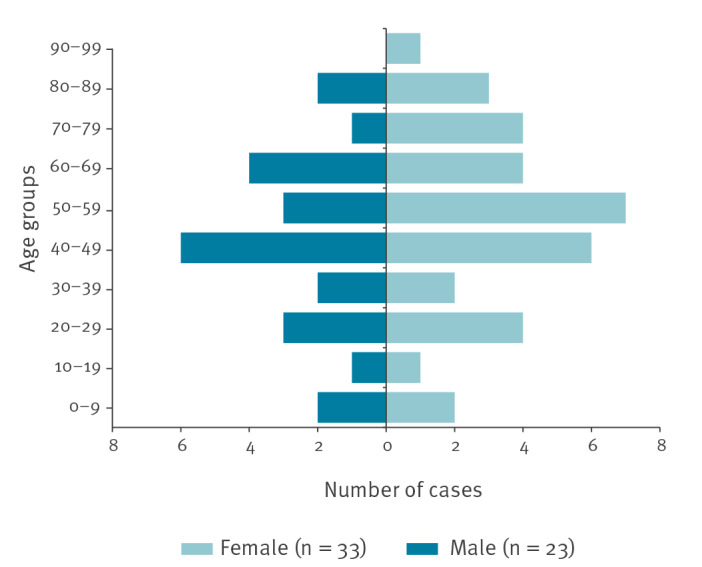
Age and sex of identified cases in the outbreak of *Salmonella* Agbeni associated with consumption of dried fruit mix, Norway, 2019 (n = 56)

**Figure 2:**
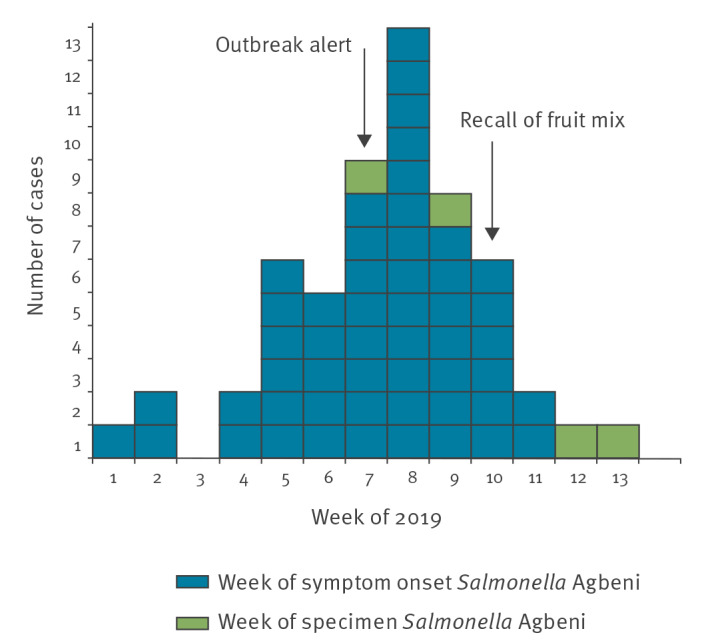
*Salmonella* Agbeni outbreak cases by week of symptom onset or week of specimen, Norway, December 2018–March 2019 (n = 56)

As of 26 April 2019, 56 confirmed cases were reported to the NIPH, 33 female and 23 male. The cases were between two and 91 years old, with a median age of 50 years (Figure 1). Date of onset was available for all but four cases, for whom only date of sampling was available. The cases reported symptom onset between 31 December 2018 (week 1) and 16 March 2019 (week 11) (Figure 2). The cases were from 13 of the 18 different counties in Norway (Akershus, Oslo, Buskerud, Østfold, Vestfold, Telemark, Vest-Agder, Rogaland, Møre and Romsdal, Trøndelag, Nordland, Troms and Finnmark).

Cases reported the following symptoms: nausea (n = 29), vomiting (n = 20), abdominal pain (n = 35), diarrhoea (n = 42) (including bloody diarrhoea (n = 13)), fever (n = 39), joint pain (n = 18) and urinary tract infection (UTI) (n = 9). In addition, several cases reported general weakness and headache. *S.* Agbeni was isolated from urine in 10 cases and from blood culture in seven cases, of which one had a UTI in addition to bacteraemia. Twenty-one cases were hospitalised, but no deaths were recorded.

On 22 February 2019, the NIPH requested information on whether other European countries had identified cases of *S.* Agbeni with the outbreak WGS type through the Epidemic Intelligence Information System (EPIS), coordinated by the European Centre for Disease Prevention and Control in Stockholm Sweden. In total, 16 European countries and the US replied to the EPIS request, but no other countries reported cases matching the Norwegian outbreak strain.

#### Trawling interviews

Twenty patients were interviewed with the *Salmonella*-specific trawling questionnaire. The interviews took place from 20 February to 5 March 2019. None of the patients reported any history of travel before symptom onset. No common restaurants/cafes or consumption of a single food item was reported by all the cases; however, 19 out of 20 cases reported intake of different mixes of nuts, dried fruits and raisins. Other common food items were spices, especially oregano (16/20), and chicken products (15/20).

### Case–control study

In total, eight cases and 31 controls participated in the case–control study. Three cases did not agree to participate in the study. Consumption of dried banana (OR: 65; 95% CI: 5–3,040) and dried mixed fruit (OR: 50; 95% CI: 3–2,437) were strongly associated with illness (Table). Other food items associated with illness were dried papaya, dried apricot and dried pineapple. No other products were associated with disease.

**Table:** Results from case–control study for *Salmonella* Agbeni outbreak, Norway, 2019 (cases = 8, controls = 31)

Exposure	Cases exposed		Controls exposed		OR (95% CI)
	n	%	n	%	
Dried banana	8	100	2	7	65 (5–3,040)^a^
Dried fruit mix	8	100	0	0	50 (3–2,437)^a^
Dried papaya	4	50	1	3	30 (2–1,523)
Dried apricot	6	75	3	10	28 (3–350)
Dried pineapple	3	38	1	3	18 (1–975)
Raisins	7	88	13	42	10 (0.99–460)
Chicken salad meat	3	38	2	7	9 (0.7–119)
Coconut chips	5	63	6	19	7 (0.93–53)
Mix of nuts and fruit	4	50	5	16	5 (0.7–38)
Mix of nuts	2	25	2	6	5 (0.3–75)
Dried mango	2	25	2	6	5 (0.3–75)
Pizza spice	3	38	6	19	3 (0.3–17)
Cashew nuts	4	50	9	29	2 (0.4–16)
Oregano	4	50	13	42	1 (0,2–9)
Peanuts	4	50	16	52	0.9 (0.2–6)
Barbecue spice	4	50	17	55	0.8 (0.1–5)
Hazelnuts	2	25	9	29	0.8 (0.07–6)
Walnuts	1	13	5	16	0.7 (0.01–9)
Chicken, fried	3	38	15	48	0.6 (0.09–4)
Cereals with nuts or dried fruit	1	13	6	19	0.6 (0.01–6)
Taco spice	4	50	21	68	0.5 (0.07–3)
Cinnamon	2	25	18	58	0.2 (0.02–2)
Almonds	1	13	15	48	0.2 (0.0–1)
Chicken fillet	1	13	21	68	0.1 (0.0–0.7)
Pepper	7	88	31	100	0 (0.0–.)
Chicken sausage	0	0	1	3	0 (0.0–.)
Herbes de Provence	0	0	5	16	0 (0.0–3)
Dried cranberries	0	0	2	6	0 (0.0–8)
Plums	0	0	2	6	0 (0.0–5)

CI: confidence interval; OR: odds ratio.

^a^ None of the controls reported consumption of dried fruit mix and all cases reported consumption of dried banana. For calculation, we assumed that individuals might not remember correctly, and moved one control as exposed and one case as unexposed for both exposures, to be able to calculate the OR.

### Additional investigations

From the electronic questionnaire with photographs of suspected food products, 18 of 27 respondents confirmed consumption of one specific dried exotic fruit mix, referred to herein as ‘Mix A’, retailed by a Norwegian distributor. Grocery store receipts from five cases confirmed purchase of this specific mix. Based on information from all available data sources, we were able to confirm that 45 out of 56 cases had consumed Mix A.

### Microbiological investigations

#### Human cases

**Figure 3:**
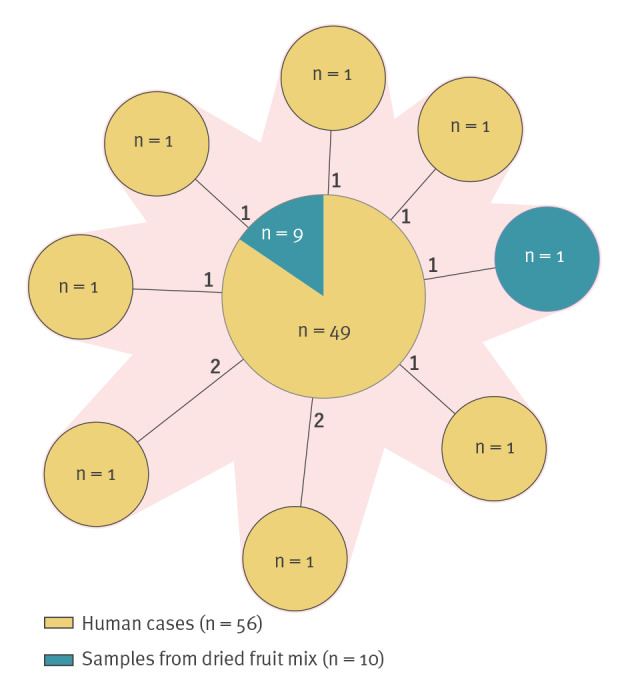
Minimum spanning tree^a^ of *Salmonella* Agbeni outbreak strains based on core genome multilocus sequence typing analysis, Norway, December 2018–March 2019

^a^ Numbers on the branches in the minimum spanning tree indicate the number of allelic differences between isolates. The number of isolates is indicated in each circle.

We identified the outbreak strain, *S*. Agbeni, ST2009 and CT2489, from all 56 cases, with ≤ 2 allelic differences between the isolates (Figure 3). One case had a dual infection with *S*. Agbeni and *S*. Wagenia.

Isolates from human cases (n = 56) are in yellow and isolates from dried fruit mix (n = 10) are in blue.

#### Food samples

Food samples were collected based on suspected products according to the trawling interviews. The food samples were provided by the cases, the supermarkets and the distributor. Samples of raisins, peanuts and different spices were all negative for *Salmonella* spp. In all, nine opened packages of Mix A from cases and two intact packages of Mix A from supermarkets were analysed. *S*. Agbeni was detected in all opened and one intact bag. In addition, another serovar of *Salmonella*, *S*. Gamaba, was detected from the two intact packages of Mix A.

WGS confirmed the isolates of *S*. Agbeni to be identical to the outbreak strain with ≤ 2 allelic differences between the isolates (Figure 3). The detected isolates of *S*. Gamaba from the two bags of Mix A were identical: ST5862 and CT2581. Additionally, one patient isolate of *S*. Gamaba that was identical to the isolates from Mix A by WGS was identified in the laboratory database at the NRL. We interviewed the patient, who reported symptom onset in early January 2019, and confirmed consumption of Mix A.

### Trace-back investigations

From information requested through RASFF, the NFSA received information about the origin of the components of Mix A. The dried fruit mix contained fruit from different suppliers: cubed pineapple and papaya from Thailand, sultanas from Turkey, sliced coconut from Ghana and banana chips from the Philippines. The mix was packed at a factory in Italy in October 2018 and exported to Norway as a ready-to-eat product. In total, 4,032 bags (400 g each) of Mix A were exported to the Norwegian distributor and then distributed to grocery stores and supermarkets.

The company in Italy had previously tested these products (17 samples), but all microbiological analyses were negative for *Salmonella* spp. Also, testing of papaya, pineapple and banana chips was performed by the suppliers and was negative. Italian food safety authorities inspected the facility and sampled sliced coconut (five samples), which were all negative for *Salmonella* spp. No other raw materials were present and available for testing at the time of inspection.

Product-tracing performed by the manufacturing company discovered that the five raw materials used for Mix A were also mixed with other ingredients in 331 lots of 66 other products that were distributed from March 2018 in six European countries. One Eastern European country received the exact same mix as Norway. However, no cases were reported from these countries through EPIS.

## Outbreak control measures

Mix A was voluntarily recalled from the market on 6 March 2019 by the Norwegian distributor (Figure 2). The dried fruit mix had been for sale in major supermarkets and grocery stores across Norway since 15 November 2018. In order to inform the public, the NIPH, the NFSA and the distributor provided information about the recall on their respective webpages. Information was also shared with national media to ensure public awareness. The recall was announced while the respondents of the case–control study were still being interviewed. Soon after the recall, we decided to stop recruitment, since the results of investigations undertaken pointed to a single product.

The NFSA also identified other products sold in Norway that contained some of the ingredients from the same batch of raw materials used for Mix A. Although the microbiological analyses of these raw materials performed by the company in Italy were negative for *Salmonella* spp., these products were also recalled on 11 March and 19 March 2019, as a precautionary measure. In total, five other dried fruit mix products were recalled. Retrospective *Salmonella* analysis of samples of these different mixes were all negative.

## Discussion

We report an outbreak of *S*. Agbeni in Norway associated with consumption of a ready-to-eat product of dried exotic fruits, referred to herein as Mix A. We were able to identify the source by combining epidemiological and microbiological methods. Cases originated from different municipalities in Norway and affected both sexes and all age groups. The arrival of new cases over time indicated a common source outbreak linked to a product with a long shelf life. This was consistent with Mix A, which had been on the market across Norway since November 2018, with the first case reported at the end of December. Microbiological analysis with high resolution typing using WGS allowed us to confirm the suspected food vehicle as the source of the outbreak, and enabled an efficient linkage of cases dispersed in time and space.

The present outbreak was characterised by an unusual, severe clinical presentation with systemic infections seen in seven, urinary tract infections in 10 and hospitalisation in 21 of the 56 cases. In general, bloodstream infections caused by non-typhoidal *Salmonella* occur in ca 6% of patients, with infants, young children, elderly and immunocompromised people being at particular risk [14,15]. It is known that different serovars of *Salmonella* vary in their ability to cause bloodstream infections and other extra intestinal diseases [15]. *S*. Agbeni is a rare serotype, and had only been reported previously from few sporadic cases in Norway. Previous outbreaks have been described from Canada in 2011, among wedding-guests; from the US in 2017, associated with pet turtles and in 2018, linked to contaminated cake mix [5-7]. Very little is described for *S*. Agbeni regarding clinical symptoms, but in the outbreak from Canada in 2011, three of eight cases were diagnosed with urinary tract infections [7]. This is in concordance with our data, where more patients than could be expected had urinary tract infections. The scientific literature reports that between 0.7 and 3.4% of non-typhoid *Salmonella* are routinely isolated from urinary tract infections [14,16,17]. This is consistent with data from the NRL in Norway, where 2.6% of the submitted non-typhoid *Salmonella* are isolated from urine (unpublished data, 2014–2018). Persons of both sexes and in all age groups were affected in this outbreak, but the majority were more than 40 years old and female. This might be of relevance for the clinical presentation, as women are more prone to UTI infection from *Salmonella* spp. than men [17]. The observed clinical symptoms and the hospitalisation proportion could thus be explained by the biological properties of the *Salmonella* serotype involved, or simply by hypothesising that women above 40 years of age are more likely to eat dried fruits than persons in other age groups. Although it is not possible to draw any conclusions about virulence and the ability to cause extra-intestinal disease for *S*. Agbeni based on the present findings, our results add to the existing knowledge about clinical manifestations for this rare *Salmonella* serotype.

Salmonellosis due to consumption of tropical fruits has previously been described in instances where raw mango, frozen mamey fruit pulp, papaya, pineapple and various types of coconut were consumed [18-20]. Exotic dried fruit products are increasing in popularity in Norway, as in the rest of Europe, as healthy alternatives to sweets [21,22]. The implicated fruit mix was packed in Italy and contained fruit originating from South East Asia and Africa. As a co-finding of the microbiological analyses of Mix A, an additional serotype, *S*. Gamaba, was detected, and in one bag both serotypes were detected. The isolate of *S*. Gamaba was linked by WGS to a historic patient isolate, where the patient confirmed intake of Mix A. In addition, one case had dual infection with *S*. Wagenia in addition to *S*. Agbeni. These findings suggest that the mix could have been contaminated with three different serotypes of *Salmonella*. It is known that *Salmonella* survives for long periods in low-moisture food products [23], and such products can therefore be regarded as products with risk for *Salmonella*. As all testing of the raw materials for Mix A was negative, we do not know where in the production process the contamination occurred. The complexity of the origin of the fruit mix and the finding of three different serovars of *Salmonella* highlight the need for strict hygiene measures when producing such ready-to-eat products.

No cases were reported from any other European countries, although dried fruits from the same batch of raw materials that were used in Mix A were distributed in other countries. There could potentially be unrecognised cases in these countries, and we can only speculate on the reasons why Norway was the only country affected. Differences in surveillance systems or laboratory routines might be an explanation, and as *S*. Agbeni is rarely encountered in Europe, isolates might not be fully identified in all laboratories [1]. In addition, WGS is not implemented for *Salmonella* typing in all countries, which might impair the comparability between European surveillance systems.

There were some limitations to this investigation. First, the passive surveillance system may have detected only a small proportion of cases, and mainly those with the most severe symptoms. Persons with simple gastroenteritis often do not seek healthcare [24]. This could influence the epidemiological determinants of infection and the demographic profile of cases. Second, retrospective interviews may lead to recall bias, especially for the participants that reported symptoms at the start of the outbreak. To overcome recall bias, we collected grocery store receipts and introduced the electronic questionnaires that included photos of specific food items. This was very valuable for the outbreak investigation, as some cases did not remember that they had consumed Mix A, but this was verified by receipts and/or the electronic questionnaire. Third, the epidemiological and microbiological investigations were carried out in parallel and did influence each other. When we started the matched case–control study, there were already media communications on the likely vehicle of the outbreak. Thus, we had to stop recruitment before a sufficient number of matched pairs were recruited, and we had to carry out an unmatched analysis of the obtained records. We are confident that our decision was correct, even though the analysis was based on few cases, because the biased results of the case–control analysis aligned very well with all the other investigations and confirmed the source by strong epidemiological association. The finding was verified by the detection of the outbreak strain in both the food product and the human cases.

This outbreak highlights dried fruits as a risk product for food-borne infections, which is of particular concern in ready-to-eat products. Public health experts, clinicians and microbiologists should be aware of this risk, especially if experiencing outbreaks with rare serotypes of *Salmonella*. The present investigation underlines the importance of a cross-sectoral outbreak investigation to identify the causative food vehicle and enable its recall, as well as the importance of EU-wide food-chain tracing. Additional investigations are needed to estimate the risk of dried fruit products for food-borne pathogens. To follow-up on this issue in Norway, the NFSA have initiated increased sampling of such products in the *Salmonella* surveillance programme for 2019 and 2020, which will provide more information about the level of contamination in these products.

## Supplementary Data

479000 Supplementary Figure S1

## References

[1] European Food Safety Authority (EFSA) and European Centre for Disease Prevention and Control. (ECDC). The European Union summary report on trends and sources of zoonoses, zoonotic agents and food-borne outbreaks in 2017. In., vol. EFSA-Q-2017-00751. Parma and Stockholm: EFSA and ECDC; 2018. Available from: https://efsa.onlinelibrary.wiley.com/doi/10.2903/j.efsa.2018.5500 10.2903/j.efsa.2018.5500 PMC7009540 32625785

[2] Lyngstad TM, Krossness MM, Salamanca BV, Lange H, Nygard K, Jore S, et al. Årsrapport 2018. Overvåking av infeksjonssykdommer som smitter fra mat, vann og dyr, inkludert vektorbårne sykdommer. [Surveillance of food- and waterborne diseases and zoonoses, including vector-borne diseases. Yearly report 2018]. Oslo: Folkehelseinstituttet; 2019. Norwegian.Available from: https://www.fhi.no/globalassets/dokumenterfiler/rapporter/2019/arsrapport-2018-overvakning-av-infeksjonssykdommer-som-smitter-fra-mat-vann-og-dyr-inkludert-vektorbarne-sykdommer.pdf

[3] Jorgensen HJ, Hauge K, Lange H, MacDonald E, Lyngstad TM, Heier B. The Norwegian Zoonoses Report 2018. Oslo: Norwegian Veterinary Institute; 2019. Available from: https://www.vetinst.no/rapporter-og-publikasjoner/rapporter/2019/the-norwegian-zoonoses-report-2018

[4] MacDonald E White R Mexia R Bruun T Kapperud G Brandal LT The role of domestic reservoirs in domestically acquired Salmonella infections in Norway: epidemiology of salmonellosis, 2000-2015, and results of a national prospective case-control study, 2010-2012. Epidemiol Infect. 2018;147:1-8. 30428947 10.1017/S0950268818002911 PMC6518537

[5] Centres for Disease Control and Prevention (CDC). Outbreak of Salmonella Infections. Atlanta: CDC; 2019 Available from: https://www.cdc.gov/salmonella/agbeni-11-18/index.html

[6] Koski L Stevenson L Huffman J Robbins A Latash J Omoregie E Notes from the Field: An Outbreak of Salmonella Agbeni Infections Linked to Turtle Exposure - United States, 2017. MMWR Morb Mortal Wkly Rep. 2018;67(48):1350. 10.15585/mmwr.mm6748a5 30521506 PMC6329487

[7] Taylor M Brisdon S Jeyes J Stone J Embree G Paccagnella A Salmonella enterica serovar Agbeni, British Columbia, Canada, 2011. Emerg Infect Dis. 2012;18(9):1542-3. 10.3201/eid1809.120008 22932699 PMC3437710

[8] Grimont PAD, Weill FX. Antigenic formulae of the *Salmonella* serovars. Paris: Institut Pasteur and World Health Organization Collaborating Centre for Reference and Research on Salmonella; 2007. Available from: https://www.pasteur.fr/sites/default/files/veng_0.pdf

[9] Achtman M Wain J Weill F-X Nair S Zhou Z Sangal V Multilocus sequence typing as a replacement for serotyping in Salmonella enterica. PLoS Pathog. 2012;8(6):e1002776. 10.1371/journal.ppat.1002776 22737074 PMC3380943

[10] Alikhan N-F Zhou Z Sergeant MJ Achtman M. A genomic overview of the population structure of Salmonella. PLoS Genet. 2018;14(4):e1007261. 10.1371/journal.pgen.1007261 29621240 PMC5886390

[11] Siira L Naseer U Alfsnes K Hermansen NO Lange H Brandal LT. Whole genome sequencing of *Salmonella* Chester reveals geographically distinct clusters, Norway, 2000 to 2016. Euro Surveill. 2019;24(4):1800186. 10.2807/1560-7917.ES.2019.24.4.1800186 30696528 PMC6352000

[12] NordVal International (NMKL). Salmonella. Påvisning i livsmedel. [Salmonella. Detection in foods]. Bergen: NMKL; 1999. Swedish. Available from: https://www.nmkl.org/index.php/nb/webshop/item/salmonella-pavisning-i-livsmedel-nmkl-71-5-utg-1999

[13] Standard Norge. Microbiology of the food chain - Horizontal method for the detection, enumeration and serotyping of *Salmonella* - Part 1: Detection of Salmonella spp. (ISO 6579-1:2017). Oslo: Standard Norge; 2017. Available from: https://www.standard.no/no/Nettbutikk/produktkatalogen/Produktpresentasjon/?ProductID=919924

[14] Vugia DJ Samuel M Farley MM Marcus R Shiferaw B Shallow S Invasive Salmonella infections in the United States, FoodNet, 1996-1999: incidence, serotype distribution, and outcome. Clin Infect Dis. 2004;38(s3) Suppl 3;S149-56. 10.1086/381581 15095184

[15] Crump JA Sjölund-Karlsson M Gordon MA Parry CM. Epidemiology, Clinical Presentation, Laboratory Diagnosis, Antimicrobial Resistance, and Antimicrobial Management of Invasive Salmonella Infections. Clin Microbiol Rev. 2015;28(4):901-37. 10.1128/CMR.00002-15 26180063 PMC4503790

[16] Zaidenstein R Peretz C Nissan I Reisfeld A Yaron S Agmon V The epidemiology of extraintestinal non-typhoid Salmonella in Israel: the effects of patients’ age and sex. Eur J Clin Microbiol Infect Dis. 2010;29(9):1103-9. 10.1007/s10096-010-0968-1 20535625

[17] Abbott SL Portoni BA Janda JM. Urinary tract infections associated with nontyphoidal Salmonella serogroups. J Clin Microbiol. 1999;37(12):4177-8. 10.1128/JCM.37.12.4177-4178.1999 10565958 PMC85919

[18] Strawn LK Schneider KR Danyluk MD. Microbial safety of tropical fruits. Crit Rev Food Sci Nutr. 2011;51(2):132-45. 10.1080/10408390903502864 21328109

[19] Luna S Taylor M Galanis E Asplin R Huffman J Wagner D Outbreak of Salmonella Chailey Infections Linked To Precut Coconut Pieces - United States and Canada, 2017. MMWR Morb Mortal Wkly Rep. 2018;67(39):1098-100. 10.15585/mmwr.mm6739a5 30286052 PMC6171899

[20] Gibbs R Pingault N Mazzucchelli T O’Reilly L MacKenzie B Green J An outbreak of Salmonella enterica serotype Litchfield infection in Australia linked to consumption of contaminated papaya. J Food Prot. 2009;72(5):1094-8. 10.4315/0362-028X-72.5.1094 19517740

[21] Statistisk sentralbyrå (SSB). Forbrukerundersøkelsen. [The consumer survey]. Oslo: SSB; 2013. Norwegian. Available from: https://www.ssb.no/statbank/table/10249/tableViewLayout1/

[22] Centre for the Promotion of Imports from developing countries (CBI). Exporting processed fruit and vegetables and edible nuts to Europe. The Hague: CBI; 2020. Available from: https://www.cbi.eu/market-information/processed-fruit-vegetables-edible-nuts

[23] Beuchat LR Mann DA. Survival of salmonella on dried fruits and in aqueous dried fruit homogenates as affected by temperature. J Food Prot. 2014;77(7):1102-9. 10.4315/0362-028X.JFP-13-549 24988015

[24] Kuusi M Aavitsland P Gondrosen B Kapperud G. Incidence of gastroenteritis in Norway--a population-based survey. Epidemiol Infect. 2003;131(1):591-7. 10.1017/S0950268803008744 12948356 PMC2869997

