# Supplementary material for: Exotic dried fruits caused Salmonella Agbeni outbreak with severe clinical presentation, Norway, December 2018 to March 2019
Source: Euro Surveill. 2021 Apr 8;26(14):2000221. doi: 10.2807/1560-7917.ES.2021.26.14.2000221 (PMC8034060; doi:10.2807/1560-7917.ES.2021.26.14.2000221)

This supplementary material is hosted by *Eurosurveillance* as supporting information alongside the article [Exotic dried fruits caused *Salmonella* Agbeni outbreak with severe clinical presentation, Norway, December 2018 to March 2019] on behalf of the authors who remain responsible for the accuracy and appropriateness of the content. The same standards for ethics, copyright, attributions and permissions as for the article apply. Supplements are not edited by Eurosurveillance and the journal is not responsible for the maintenance of any links or email addresses provided therein.

Supplementary Figure S1. Photograph of Mix A, the suspected dried exotic fruit mix product packaging

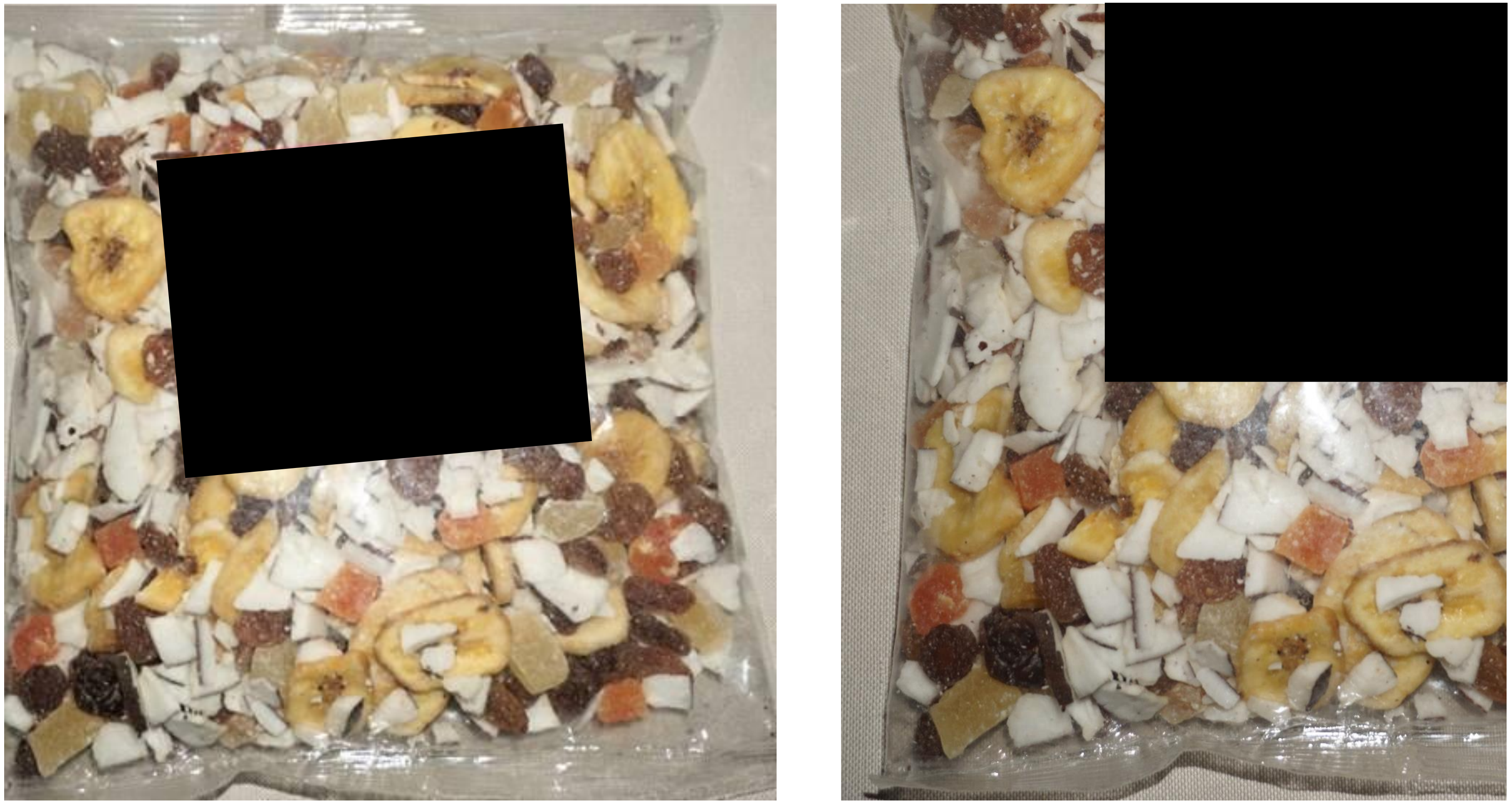

Supplement: Supplementary Figure S1 [file 2000221_SupplementaryFigure.pdf]
